# Highly Integrated Elastic Island-Structured Printed Circuit Board with Controlled Young’s Modulus for Stretchable Electronics

**DOI:** 10.3390/mi11060617

**Published:** 2020-06-25

**Authors:** Duho Cho, Junhyung Kim, Pyoenggeun Jeong, Wooyoung Shim, Su Yeon Lee, Youngmin Choi, Sungmook Jung

**Affiliations:** 1 Division of Advanced Materials, Korea Research Institute of Chemical Technology (KRICT), 141 Gajeongro, Daejeon 305-600, Korea; chlarm16@krict.re.kr (D.C.); kjh2@krict.re.kr (J.K.); gunbiz@krict.re.kr (P.J.); sylee@krict.re.kr (S.Y.L.); 2 Department of Materials Science & Engineering, Yonsei University, Yonsei-ro Seodaemun-gu, Seoul 03722, Korea; wshim@yonsei.ac.kr; 3 Department of Chemical Convergence Materials, Korea University of Science and Technology (UST), 217 Gajeongro, Yuseong-gu, Daejeon 305-350, Korea

**Keywords:** stretchable printed circuit board (PCB), substrate, island structure, 3D nozzle printing, degree of integration

## Abstract

A stretchable printed circuit board (PCB), which is an essential component of next-generation electronic devices, should be highly stretchable even at high levels of integration, as well as durable under repetitive stretching and patternable. Herein, an island-structured stretchable PCB composed of materials with controlled Young’s modulus and viscosity by adding a reinforcing agent or controlling the degree of crosslinking is reported. Each material was fabricated with the most effective structures through a 3D printer. The PCB was able to stretch 71.3% even when highly integrated and was patterned so that various components could be mounted. When fully integrated, the stress applied to the mounted components was reduced by 99.9% even when stretched by over 70%. Consequently, a 4 × 4 array of capacitance sensors in a stretchable keypad demonstration using our PCB was shown to work, even at 50% stretching of the PCB.

## 1. Introduction

Shape-transformable electronics are in the spotlight. Currently, only flexible products have been commercialized, but in the future, this trend will be replaced by stretchable electronics that conform to any surface topology and are mechanically insensitive to fatigue strain. This would greatly expand their field of application. For example, a stretchable healthcare device could be deformed to fit the human body [1,2] to provide accurate diagnosis and therapy. This could be expanded to other fields such as renewable energy [3,4], robotics [5,6], the military [7,8], and lighting [9,10]. However, not all of the components used in current shape-transformable electronics are stretchable, so performance deteriorates markedly when stretched [11].

With this in mind, an island-structured printed circuit board (iPCB) is proposed [12]. An iPCB is composed of rigid sections where rigid components (transistors, light-emitting diodes (LEDs), photovoltaics, resistors, etc.) are mounted on a board, and stretchable sections that connect them [13,14,15,16]. For example, to make the Arduino Uno elastic by means of an iPCB, as shown in Figure S1, the section where the electronic components are mounted is rigid, and the remaining sections are stretchable. The rigid section is made of materials with a high Young’s modulus, which should not be affected by stretching. They are also patternable. The stretchable sections are made of a low Young’s modulus material with high elasticity and durability, even when subjected to repeat stretching. However, the reported iPCB research results [17,18,19] highlight low pattern conformity of the rigid sections and stress concentration at the interface between the rigid and the soft sections due to the large Young’s modulus difference between them [20,21]. Subsequently, the elongation of the entire substrate was lower than 20%, the durability of repeated elongation was poor, and the degree of integration was low. The degree of integration means that the ratio of the area occupied by the rigid section in the iPCB and is referred to as the *R* ratio in this paper.

In this work, three polydimethylsiloxane (PDMS)-based elastomers with different Young’s modulus are proposed for a stretchable iPCB. For the solid interface, all sections should be constructed from an identical material, showing excellent adhesion without additional bonding materials. PDMS was selected because it is biocompatible enough to receive FDA approval and its properties, such as stretchability, modulus, and adhesiveness, can be adjusted according to the manufacturing method. A material for the soft section should have a Young’s modulus lower than 0.05 MPa and excellent durability against repeated stretching [22]. The material for the rigid section should have a Young’s modulus of 100 times that of the soft section. With this approach, the stress caused by stretching can be reduced by 99.9%.

The rigid material was patterned using a 3D nozzle printing process [23,24,25,26,27,28], making it possible to print the rigid section according to the shape and size of each electronic device. To alleviate the rapid change in Young’s modulus, an intermediate section of moderate modulus was added to the interface between the rigid section and the soft section. With this rigid–intermediate–soft (RIS) substrate, we maximized the stretchability and the degree of integration of the iPCB. To demonstrate this, we fabricated a stretchable keypad with a 4 × 4 array of touch sensors printed on the iPCB. Then, we proved that the keypad worked normally even after 50% stretching of the iPCB.

## 2. Materials and Methods

### 2.1. Preparation of Rigid–Intermediate–Soft (RIS) Substrate Paste

The rigid section paste was prepared by mixing 3.0 g of polydimethylsiloxane (PDMS, 10:1 mixture of a prepolymer: curing agent, Sylgard 184, Dow Corning, Midland, MI, USA), 4.5 g of glass fiber (GF) (milled fiber glass 50 um, Fiberman, Burnaby, BC, Canada), and 0.3 g of hexane (Daejung Chemicals & Materials Co., Siheung-si, Gyeonggi-do, Korea) using a Thinky Mixer (ARE-310, Japan) for 3 min, deformed for 1 min, and then mixed for 1 min. The intermediate section paste was prepared by mixing high-viscosity PDMS (10:1 mixture of a prepolymer:curing agent, Sylgard 186, Dow Corning) using a Thinky Mixer for 3 min, deformed for 1 min, and then mixed for 1 min. The soft section solution was prepared by mixing PDMS (20:1, Sylgard 184) and a reverse-micelle-induced (RMI) solution. First, a solution for the aqueous core of the RMI was prepared by stirring 1.0 mL of ethanol (95%, Samchun Chemical) and 0.1 g of 5-amino-1-pentanol (95%, Sigma-Aldrich, St. Louis, MO, USA) [22]. Then, 0.1 mL of the solution was mixed with 5.0 mL of octane (95%, Samchun Chemical Co., Seoul, Korea) and 1.0 mL of 1-octanol (99%, Sigma-Aldrich). Finally, 1.5 mL of the RMI solution was mixed with 10.0 g of PDMS (20:1, Sylgard 184).

### 2.2. RIS Substrate Formation and Patterning Methods

The rigid section paste and intermediate section paste were printed on Teflon tape using the ShotMaster 200DS.350PC (Musashi Engineering, Inc., Mitaka City, Tokyo, Japan). A tapered tip with a diameter of 200 μm (RT Corp., Laramie, WY, USA, 7018417) and a 5 cc syringe barrel (RT Corp.) were used. The printed pastes were cured in an oven at 80 °C for 30 min. Then, the soft section was poured and cured in an oven, at 50 °C for 4 h, and 80 °C for 14 h.

### 2.3. Characterization of the RIS Substrate

To measure the RIS substrate’s Young’s modulus, strain–stress curve, and durability, a force gauge (M7-2, Mark-10, Mark-10 Corporation, Copiague, NY, USA) and a motorized test stand (ESM 303, Mark-10, USA) were used. To quantify the adhesion force between each section, we use a customized adhesion tester consisting of the above force gauge and a motorized test stand controlled using motion control software. The adhesion force was measured by following the standard test procedure (ASTM D3330). To measure the electrical properties during stretching, a uniaxial stretching machine (Jaeil Optical system), a digital multisource meter (2450, Keithley Instruments Inc., Cleveland, OH, USA), a step motor controller (NMC-201N, COPIA), liquid metal (Gallium-Indium eutectic, Sigma-Aldrich), and copper wire were used. The morphology was characterized by means of scanning electron microscopy (SEM, Quanta FEG 650, FEI. Ltd., Natural Bridge Station, VA, USA) and optical microscopy (OM, BX51).

### 2.4. Preparation of the Double Layer Stretchable Keypad

A stretchable conductive paste was prepared by mixing 1.6 g of silver flakes (DSF-500MWZ-S, Daejoo Electronic Materials Co., Gyeonggi-do, Korea), 0.6 g of PDMS (10:1, Sylgard 184):silicone oil (200F, Saehan Silichem corp., Gyeonggi, Korea):octane solution (2.5 g:0.5 g:2.0 g) using a Thinky Mixer (ARE-310, Japan) for 3 min, deformed for 1 min, and then mixed for 1 min. A conductive paste for via-hole filling was prepared by mixing 1.65 g of silver flakes, 0.6 g of PDMS:silicone oil:octane solution (2.5 g:0.5 g:3.0 g) using a Thinky Mixer (ARE-310, Japan) for 3 min, deformed for 1 min, and then mixed for 1 min.

**Figure 1:**
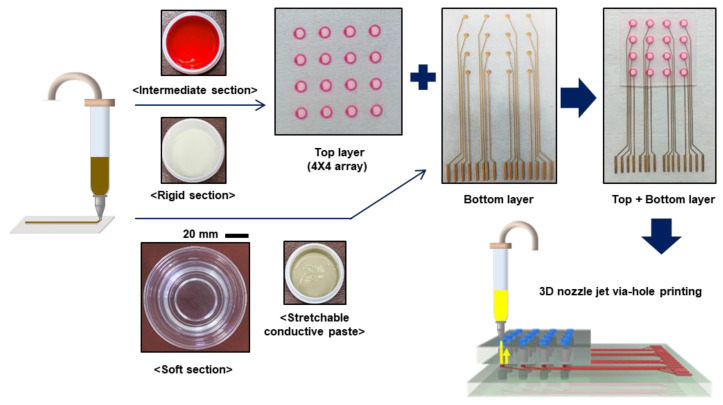
Schematic illustration of the manufacturing process for the double layer stretchable keypad.

Figure 1 illustrates the overall process for preparing the keypad. The stretchable conductive pastes were printed on a silicone–rubber substrate (Ecoflex 00-30) using the ShotMaster 200DS.350PC (Musashi Engineering). A general-purpose tip with a diameter of 150 μm (RT Corp., 7018424) and a 5-cc syringe barrel (RT Corp.) were used. The printed pattern was cured in an oven at 50 °C for 40 min, 80 °C for 1 h, and then 130 °C for 3 h. Via holes were formed on the rigid section of the top layer using a punch (1 mm, Miltex). Then, the top layer was attached to the bottom layer (see Figure 1). The via-hole conductive paste was printed on the via holes using a Shot mini 200SX (Musashi Engineering, Japan). A general-purpose tip with a diameter of 150 μm (RT Corp., 7018424) and a 5 cc syringe barrel (RT Corp.) were used. The printed pattern was cured in an oven at 50 °C for 40 min, 80 °C for 1 h, and 130 °C for 3 h.

### 2.5. Characterization of the Double-Layer Stretchable Keypad

The terminals of the printed pattern of the keypad and the copper wires were connected using liquid metal (Gallium-Indium eutectic, Sigma-Aldrich) and a NaOH 3% diluted solution (NaOH, 99%, Sigma-Aldrich:DI water 3 wt%) [29]. The connected wires were fixed using Ecoflex (00-30), linking the keypad to a microcontroller unit (Arduino Leonardo), which could detect and send changes in the capacitance signals to a PC. A custom developed Arduino program analyzed the output signals and activated specific keypad values. When the value of each channel of the keypad increased above a threshold, the PC executed a corresponding, specific value. If the value did not exceed the threshold, a keypad ‘release’ was performed.

## 3. Results

### 3.1. Rigid, Intermediate, and Soft Materials for Stretchable Island-Structured Printed Circuit Board (iPCB)

#### 3.1.1. Rigid Section

**Figure 2:**
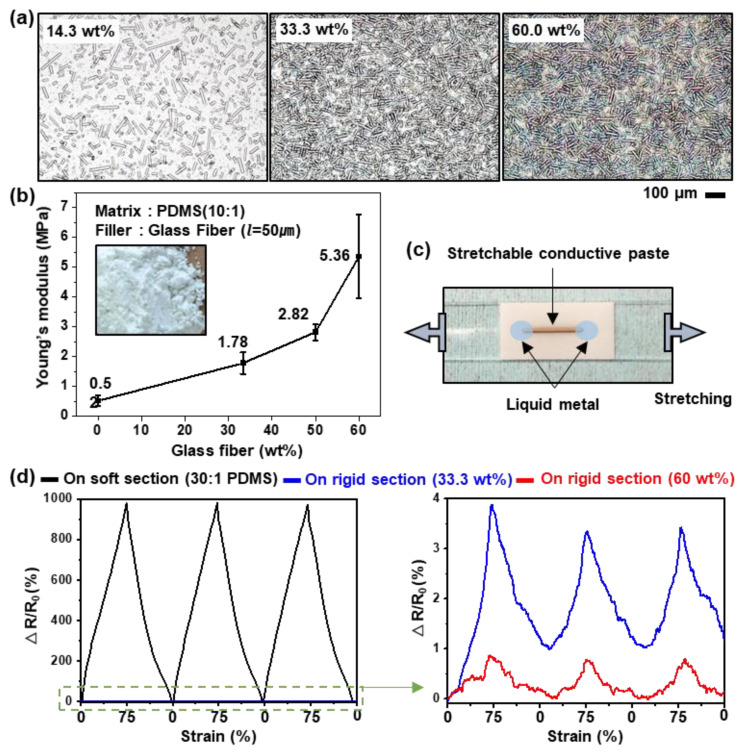
(**a**) Optical microscopy images of the rigid section film 14.3, 33.3, and 60 wt% glass fiber (GF), (**b**) and their Young’s modulus. (**c**) Image of the printed stretchable conductive paste on the rigid section. (**d**) Resistance changes of the printed stretchable conductive paste on the soft section (**left**) and the rigid section made with 33.3 and 60 wt% GF (**right**) according to tensile strain (75%, 3 cycles).

**Figure 3:**
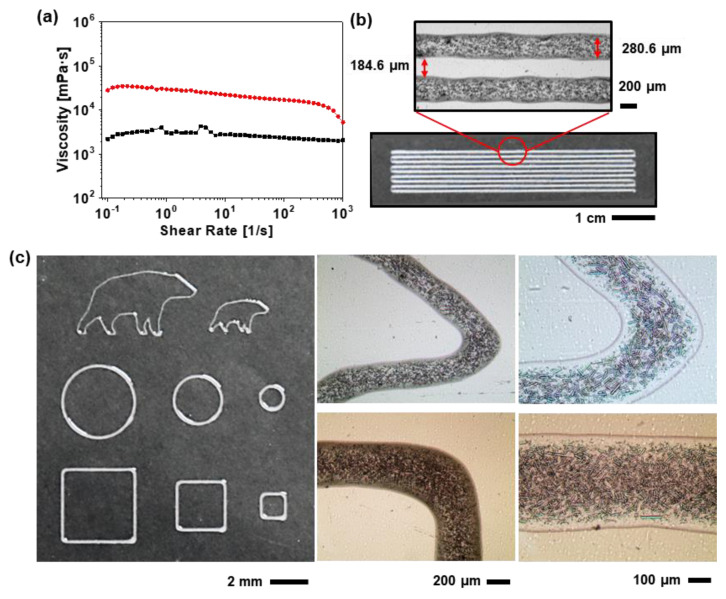
(**a**) Viscosity of the rigid part (with 60 wt% GF) with solvent (Black) and without solvent (Red). (**b**) Image of printed rigid part through nozzle jet printing. Inset shows a magnified optical microscopy image. (**c**) Image of variously patterned rigid part through nozzle jet printing. Inset shows a magnified optical microscopy image.

The solidified PDMS was made by crosslinking siloxane oligomers and siloxane hydrides with a Pt-based catalyst. The PDMS could be made harder with more crosslinking by adjusting the ratio of the catalyst or the reaction temperature, but it is difficult to achieve a high Young’s modulus so that there is no change in area during iPCB stretching. 1D GF, which is composed of SiO_2_ and is well dispersed in the PDMS, was added as a reinforcement agent to the PDMS for a high Young’s modulus. As shown in Figure 2a and Figure S2, the glass fibers added to the PDMS were evenly distributed, and as the weight ratio increased, so did the density. When GF was added in excess of 60 wt%, partial aggregation of the GF was observed using an optical microscope. Agglomerated fillers caused clogging during the nozzle jet printing process, so the paste with more than 60 wt% GF was excluded. The addition of 33.3, 50.0, and 60.0 wt% of GF to the PDMS with 0.52 MPa Young’s modulus increased the Young’s modulus to 1.78, 2.82, and 5.36 MPa, respectively (Figure 2b). In order to determine whether this was suitable for use as a rigid section, a film with a rigid section containing 33.3 or 60.0 wt% of GF and a soft section made of 30:1 PDMS was produced in a 1:1 area ratio. Thereafter, a stretchable conductive paste whose resistance was changed by strain was printed on the rigid section, and then stretched 75% to measure the resistance change (Figure 2c). For comparison, a film comprising only soft sections was tested in the same way. Using the soft section alone, the resistance increased by approximately 1000% when stretched at 75%, whereas in the rigid section containing 33.3 and 60.0 wt% GF, the resistance increased by approximately 4% and 1%, respectively (Figure 2d, Supporting Movie S1). Therefore, in subsequent experiments, a PDMS paste containing 60 wt% of GF was used to produce the rigid sections. This paste could be printed at approximately 280 µm line width using the nozzle jet printer (Figure 3). The viscosity of the paste was 10,000 Pa∙S or more, suitable for 3D printing, and when 10% of hexane was added, the viscosity decreased, making it suitable for producing flattened rigid sections.

#### 3.1.2. Soft Section

**Figure 4:**
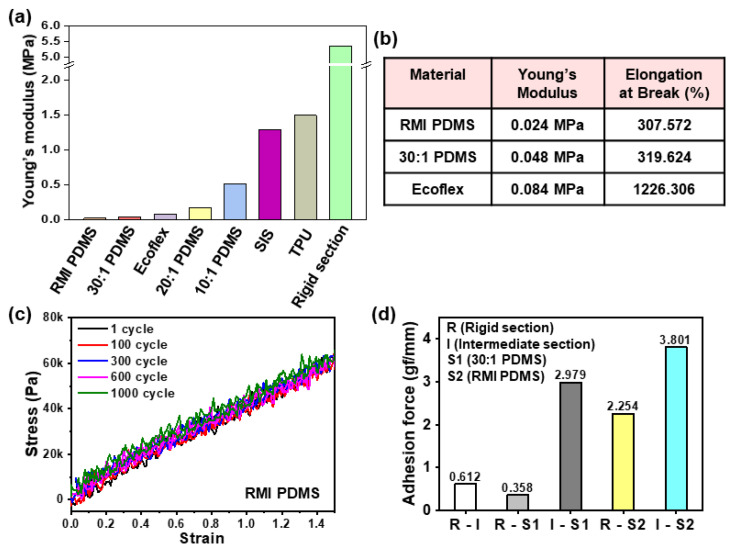
(**a**) Young’s modulus of various materials for the soft section. (**b**) Young’s modulus and elongation at the break of reverse-micelle-induced (RMI) polydimethylsiloxane (PDMS), 30:1 PDMS, and Ecoflex. (**c**) The stress–strain curves of RMI PDMS during 150% stretching over 1000 cycles. (**d**) Adhesion force of the soft sections (RMI PDMS and PDMS 30:1) with the rigid section and the intermediate section.

Since the rigid section was not stretched during iPCB stretching, the soft section had to stretch instead, and the higher the degree of integration, the higher the stress. For highly integrated iPCBs where rigid sections occupy 80% of the area, to stretch by 40%, the soft sections must increase by 200% (Figure S3). Therefore, the soft section should not only have a low Young’s modulus, but also high stretchability, repeatable stretch durability, and high adhesion. Figure 4a shows the Young’s modulus of candidate materials that could be used for soft sections. Ecoflex, PDMS, and RMI PDMS have low Young’s modulus values of 84, 48, and 24 kPa, respectively, and they are capable of stretching 1226.3%, 319.6%, and 307.6%, respectively (Figure 4b). The stress–strain curve of the RMI film hardly changed even after 1000 repeated stretching cycles, and it had the best adhesion force to the materials used in the rigid and intermediate sections (Figure 4c,d). Therefore, in subsequent experiments, RMI PDMS was used for the production of the soft sections (Figure S4).

#### 3.1.3. Intermediate Section

Due to the high adhesion force and low Young’s modulus of the soft section, the iPCB with an *R* ratio of 33% could be stretched by up to 150%. However, as the degree of integration increases, elongation and durability decreased, so the iPCB with an *R* ratio of 80% broke at as little as 39.6% elongation (Figure S5). It has been reported that gradually changing the Young’s modulus between the soft section and the rigid section (at the intermediate section, *I* section) relieves the stress at the interface [30]. However, the reported method had a problem in that the *I* section was attached to the outside of the substrate, so that the surface of the substrate was not flat. Considering these points, three criteria were selected for the selection of *I* section materials. First, it had to be a silicone-based elastomer for adhesion to other sections. Second, the Young’s modulus had to be an intermediate value between the rigid and soft section materials. Third, since it could be made from a high-viscosity paste, it had to be able to be located at the rigid section interface through printing.

**Figure 5:**
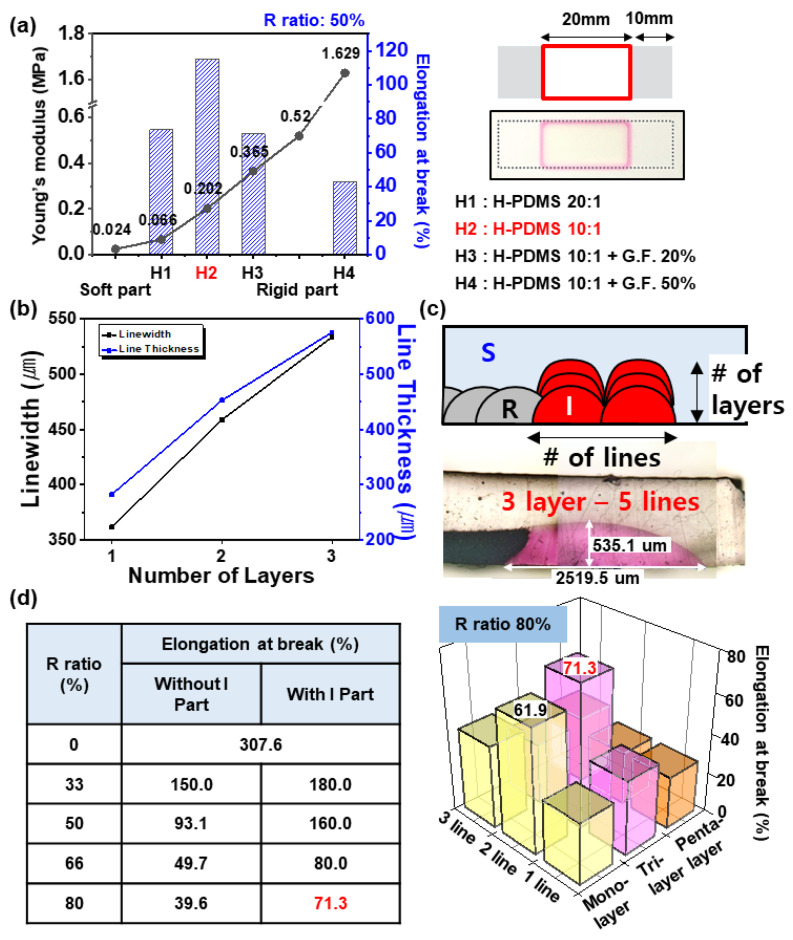
(**a**) Dependence of elongation at the break of the island-structured printed circuit board (iPCB) with an *R* ratio of 50% on the Young’s modulus of *I* section changes by adding GF or adjusting the amount of catalyst. (**b**) Change of linewidth and line thickness according to *z*-axis stacking printing. (**c**) Optical microscopy image of *z*-axis stacking printed *I* section. (**d**) Dependence of elongation at the break of the iPCB with *R* ratios of 0, 33%, 50%, 66%, and 80% according to the presence of the *I* section (**left**) and dependence of elongation at the break of the iPCB with an *R* ratio of 80% according to the 3D structure changes (**right**).

High-viscosity PDMS (H-PDMS, Sylgard 186, 65,000 mPa∙S) is a silicone-based elastomer of high viscosity (Figure S6). Moreover, it is possible to control the Young’s modulus of H-PDMS by adding GF or adjusting the amount of catalyst, so that it could have the required median Young’s modulus (Figure 5a). As a result of stretch testing the iPCBs (*R* ratio of 50%), where each H-PDMS was printed on the interface with the same thickness and linewidth (Supporting Movie S2), the iPCB made of 0.202 MPa Young’s modulus H-PDMS showed the best stretchability (115%, Figure S7). Therefore, in subsequent experiments, H-PDMS 10:1 was used to produce *I* sections. High-viscosity H-PDMS is capable of 3D printing. After printing in a straight line and moving in the *z*-axis and stacking them, the line width of a single line increases from 361.6 to 533.8 um, and the line thickness increases from 282.2 to 576.1 um (Figure 5b). In addition, as shown in Figure 4c, the linewidth of the *I* section can be freely increased by printing multiple 3D printed lines. As a result of stretching tests of the iPCBs with *I* sections of various linewidth and thickness, it was confirmed that an optimum effect was obtained at a linewidth of 1530.33 um and a line thickness of 349.33 um (tri-layer, 2 lines). For an iPCB with an *R* ratio of 80%, it can be stretched up to 71.3%, which increases the stretchability by 80% compared to an iPCB without the *I* section (Figure 5d, Table 1).

### 3.2. Stretchable Electronics Demonstration using Stretchable iPCB

**Figure 6:**
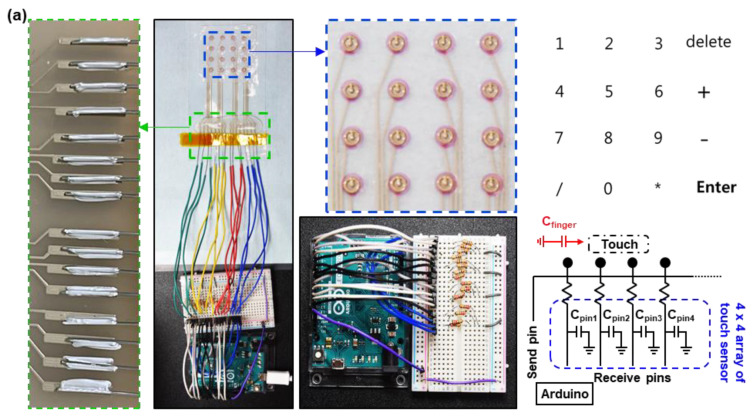

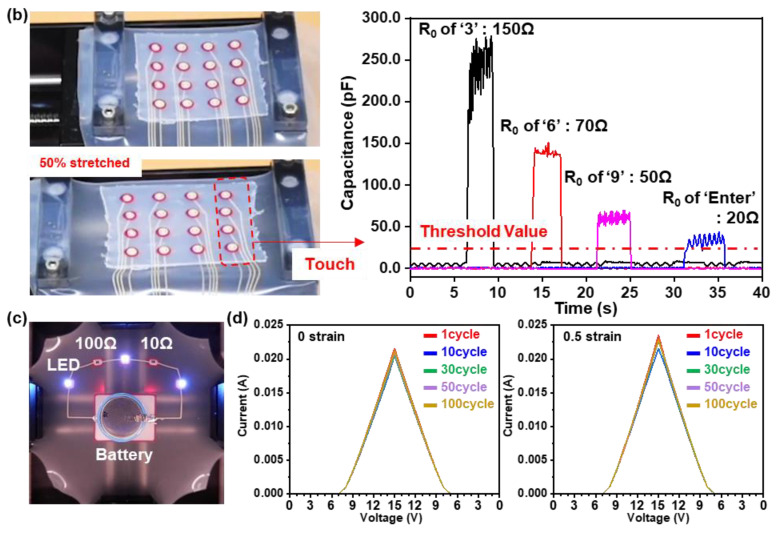
Stretchable keypad demonstration. (**a**) Photograph of the system. 4 × 4 array of touch sensors printed on the iPCB made with rigid–intermediate–soft (RIS) materials and connected to an Arduino (**left**). Keypad configuration (**right top**) and a circuit diagram of the system (**right bottom**). (**b**) Optical image of 50% stretched stretchable keypad (**left**). Change in capacitance during touch of ‘3’, ‘6’, ‘9’, and ‘Enter’ on the keypad (**right**). (**c**) Image of a stretchable iPCB with electrical components (chip LEDs, resistors, and battery). (**d**) Current output signals from the device at 0 and 0.5 strain.

To demonstrate the applicability of the iPCB with rigid/intermediate/soft materials in stretchable electronics, we fabricated a stretchable keypad, which had a 4 × 4 array of capacitance sensors, connected to a microcontroller unit (MCU, Arduino Uno). Figure 6a shows the overall layout of the stretchable keypad system. The keypad consisted of 10 numeric keys, four arithmetic keys, a delete, and an enter key. The capacitance sensors on the rigid section of the top layer were connected to the bottom layer through the via hole and the MCU by using stretchable conductive paste or liquid metal (Figure S8). The MCU continuously measured and displayed the changes in the capacitance value of each channel and carried out a command when the signal exceeded a threshold value. Figure 6b shows the 50% stretched keypad and the value changes when the ‘3’, ‘6’, ‘9’, and ‘Enter’ keys were touched. Since the length of the electrode connecting each sensor channel to the MCU was different, the initial resistance value and the amount of change in the signals were different. However, all channels exceeded the threshold value when touched, so there was no problem in performing the keypad operation (Supporting Movie S3). The applicability of the iPCB was further demonstrated using rigid components. We fabricated an iPCB consisting of a battery (15 V, 1000 mAh), three blue light-emitting diodes (LEDs), two resistors (10 Ω and 100 Ω), and the stretchable conductive paste for connecting the battery to the LEDs. Liquid metal was used to connect the LEDs and battery to the printed stretchable electrode. The brightness of the LEDs was barely affected by the strain on the iPCB (Figure 6c, Figure S9, and Supporting Movie S4). After monitoring the current–voltage characteristics of the circuit with digital multi-source meter (2400, Keithley Instruments Inc.), it was confirmed that the characteristics hardly changed after 100 cycles of 0.5 strain (Figure 6d). For the application design of the iPCB, the dielectric constant and loss tangent of each material constituting the PCB were measured by using Network analyzer (E4991A). The dielectric constant and loss tangent values measured in the range of 30 MHz to 2 GHz are plotted in Figure S10. The dielectric constant did not change even at high frequencies, but the loss tangent rose sharply at 2 GHz.

## 4. Discussion

The proposed rigid, intermediate, and soft materials for a stretchable iPCB proved that 70% or more stretchability could be achieved even if the degree of integration was 80%. We defined the properties required for each section of the iPCB and developed suitable materials. All materials were constructed based on the silicone rubber but had different Young’s moduli through property modifications, such as adding a reinforcing agent or controlling the degree of crosslinking. The material for the rigid section had a very high Young’s modulus so that it did not stretch even when the iPCB was stretched. This meant that the stress applied to the mounted devices was reduced by 99.9%. Due to its high viscosity, it could be patterned in various shapes and sizes. The material for the soft section had a very low Young’s modulus, exhibiting an elongation of 300% or more, with excellent repetition elongation durability and high adhesion. The material for the intermediate had a medium Young’s modulus to relieve the concentration of stress that is known to occur at the interface between the rigid and soft sections. 3D printing was possible due to the high viscosity chosen to maximize the stretchability of the stretchable iPCB through an optimal 3D structure. Each material was effective in realizing a highly integrated stretchable iPCB, thereby advancing the realization of stretchable electronics. The required physical properties and material selection criteria of each section verified through this study may be extended to other elastomer materials such as styrene–butadiene–styrene (SBS) or polyurethane (PU).

## Figures and Tables

**Table 1:** Comparison of different stretchable iPCB for stretchable electronic devices. RMI: reverse micelle induced, PDMS: polydimethylsiloxane.

Rigid Material	Soft Material	*R* Ratio (%)	Elongation at Break (%)	Ref.
Hard PDMS (Photolithography)	Soft PDMS	50	70	19
PDMS	UV cured PDMS	27	200	20
SU8	PDMS	33.3	20	21
Alumina-PU	PU	33.3	350	30
Glass fiber-PDMS	RMI-PDMS	50	160	This work
Glass fiber-PDMS	RMI-PDMS	80	71.3	This work

## Supplementary Materials

The following are available online at https://www.mdpi.com/2072-666X/11/6/617/s1, Figure S1: Schematic illustration of the island-structured PCB (iPCB) for the Arduino. Figure S2: Optical microscopy images of the rigid part pastes made with 14.3, 20.0, 25.0, 33.3, 50.0 and 60 wt% glass fiber. Figure S3: Dependence of stretchable part elongation on the 100% elongation of total substrate with various rigid part ratios. Figure S4: (a) The stress-strain curves of RMI PDMS, (b) Ecoflex and (c) PDMS 30:1 during 150% stretching 1000cycles. Inset shows stress-strain cuves at 1, 300, 600 and 1000cycle. Figure S5: Dependence of elongation at break of the interface between rigid part and soft part without intermediate part on rigid part ratio. Inset shows a magnified optical microscopy image of interface between rigid part and soft part. Figure S6: Rheological property of the pastes. Figure S7: Image of printed RIS substrate with rigid part ratio 33, 50, 66 and 80%. Figure S8: Schematic diagram of two layer 4 x 4 capacitance sensor array (top), and its photograph (bottom). Figure S9: Images of a iPCB with resistors, LEDs, and battery at rest (top) and under 0.5 strain (bottom). Figure S10: Dielectric constant (left) and loss tangent (right) change of ‘R’, ‘I’, and ‘S’ materials according to frequency. Supporting Movie S1: Resistance change of strain sensor on the Rigid part. Supporting Movie S2: Nozzle jet printing. Supporting Movie S3: Google calculator demo. Supporting Movie S4: Stretchable electronics demo.
