# Supplementary material for: Highly Integrated Elastic Island-Structured Printed Circuit Board with Controlled Young’s Modulus for Stretchable Electronics
Source: Micromachines (Basel). 2020 Jun 25;11(6):617. doi: 10.3390/mi11060617 (PMC7344695; doi:10.3390/mi11060617)
Supplement: Supplementary file 1 [file micromachines-11-00617-s001.zip › micromachines-808341-supplementary-for final/micromachines-808341-for final-supplementary.docx]

Supporting Information

Highly Integrated Elastic Island-Structured Printed Circuit Board with Controlled Young’s Modulus for Stretchable Electronics

Duho Cho, Junhyung Kim, Pyoenggeun Jeong, Wooyoung Shim, Su Yeon Lee, Youngmin Choi and Sungmook Jung


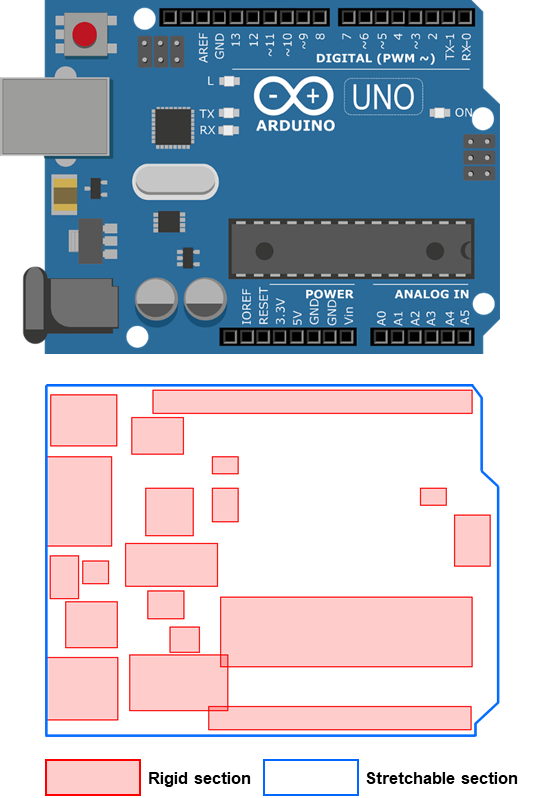


**Figure S1.** Schematic illustration of the island-structured PCB(iPCB) for the Arduino.


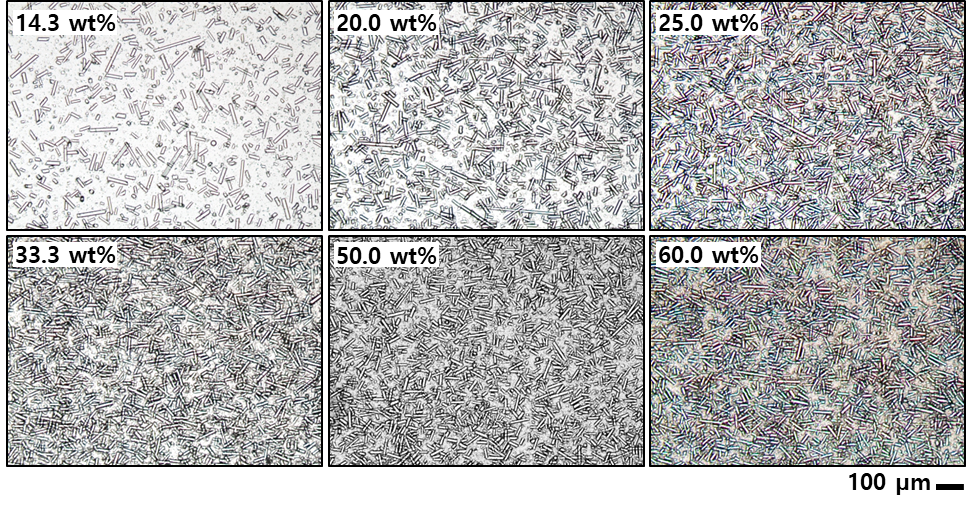


**Figure S2.** Optical microscopy images of the rigid part pastes made with 14.3, 20.0, 25.0, 33.3, 50.0 and 60 wt % glass fiber.


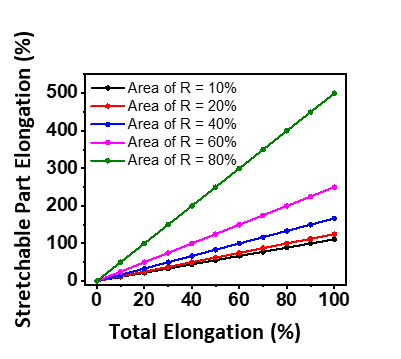


**Figure S3.** Dependence of stretchable part elongation on the 100% elongation of total substrate with various rigid part ratios.


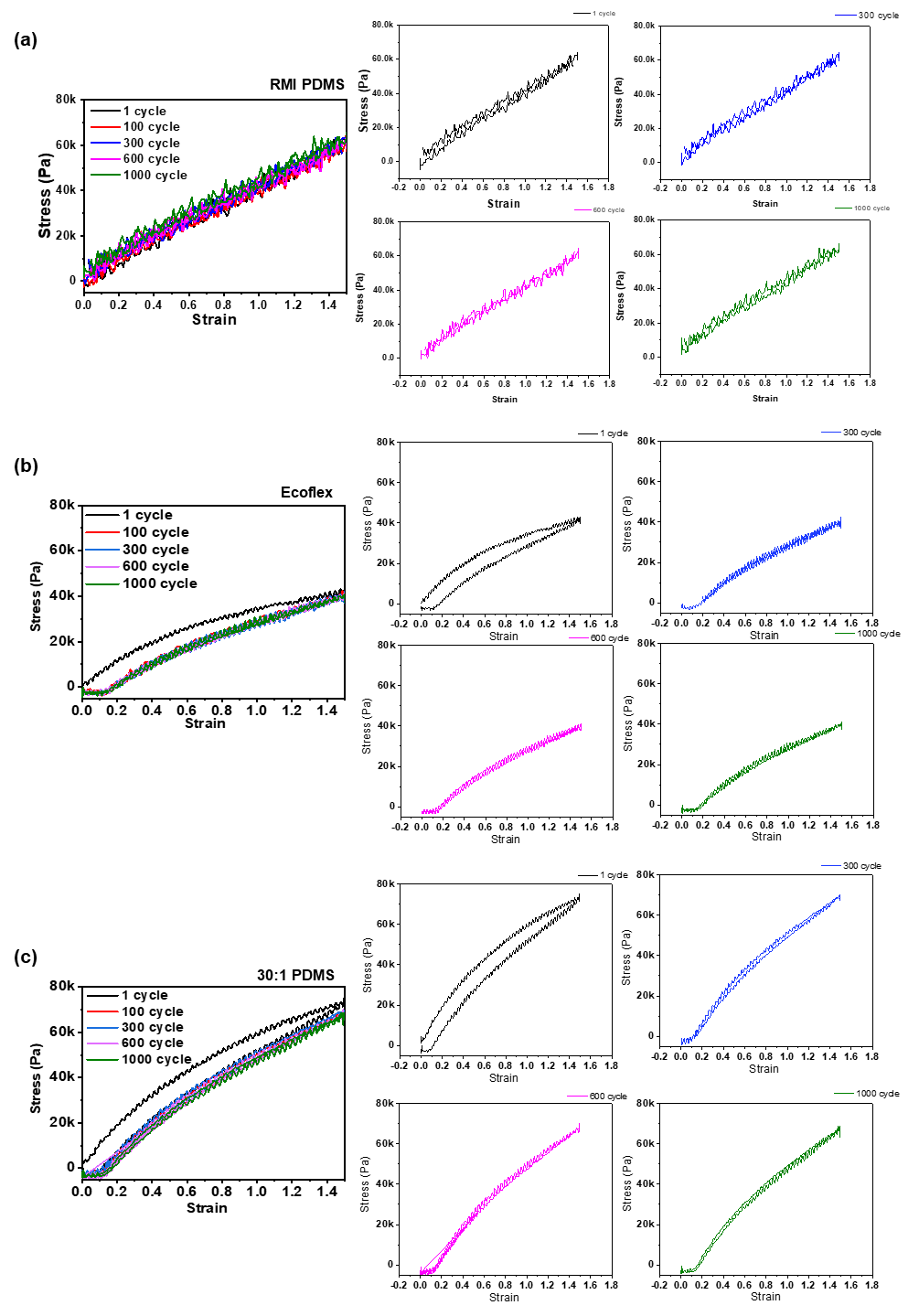


**Figure S4.** (**a**) The stress-strain curves of RMI PDMS, (**b**) Ecoflex and PDMS 30:1 during 150% stretching 1000cycles. (**c**) Inset shows stress-strain cuves at 1, 300, 600 and 1000cycle.


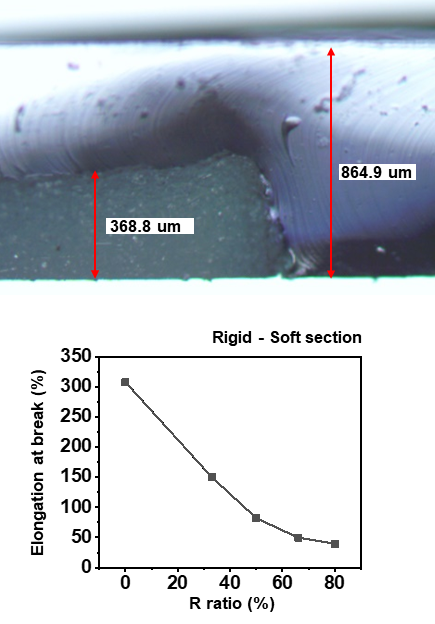


**Figure S5.** Dependence of elongation at break of the interface between rigid part and soft part without intermediate part on rigid part ratio. Inset shows a magnified optical microscopy image of interface between rigid part and soft part.

**Figure S6.** Rheological property of the pastes.


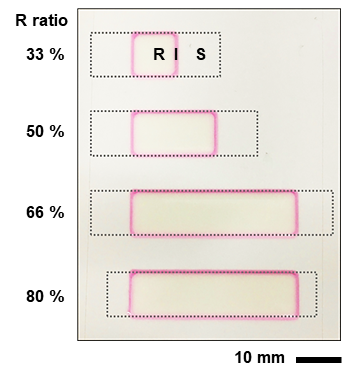


**Figure S7.** Image of printed RIS substrate with rigid part ratio 33, 50, 66 and 80%.


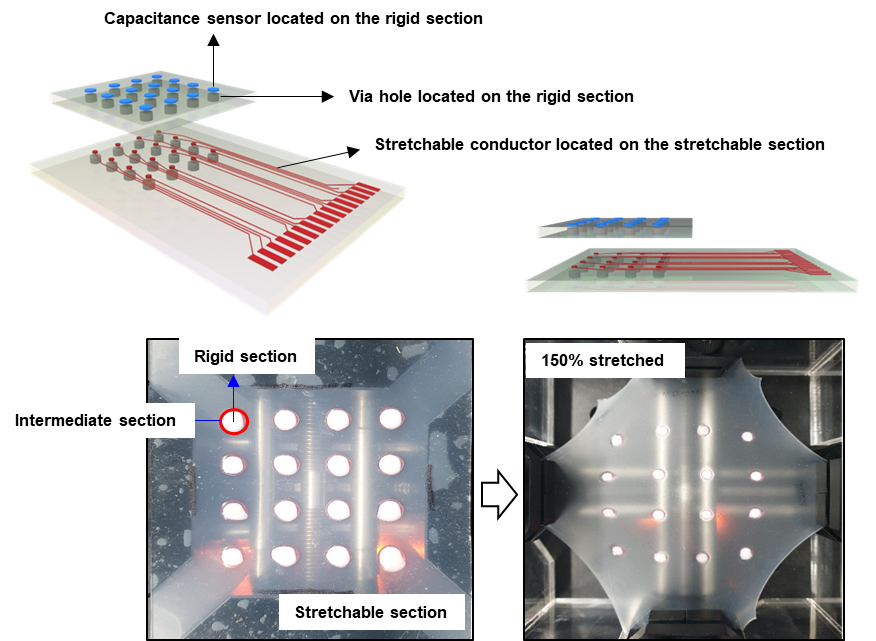


**Figure S8.** Schematic diagram of two layer 4 × 4 capacitance sensor array (**top**), and its photograph (**bottom**).


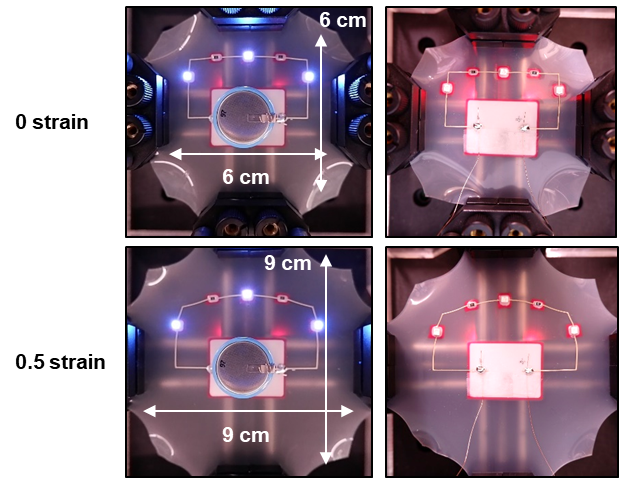


**Figure S9.** Images of a iPCB with resistors, LEDs, and battery at rest (**top**) and under 0.5 strain (**bottom**).


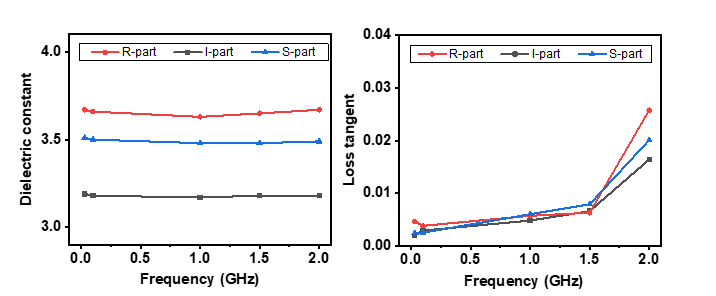


**Figure S10.** Dielectric constant (**left**) and loss tangent (**right**) change of ‘R’, ‘I’, and ‘S’ materials according to frequency**.**

| 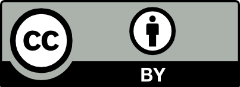 | © 2020 by the authors. Submitted for possible open access publication under the terms and conditions of the Creative Commons Attribution (CC BY) license (http://creativecommons.org/licenses/by/4.0/). |
| --- | --- |
